# Lymphoid Tumors of *Xenopus laevis* with Different Capacities for Growth in Larvae and Adults

**DOI:** 10.1155/1994/37392

**Published:** 1994

**Authors:** Jacques Robert, Chantal Guiet, Louis Du Pasquier

**Affiliations:** Basel Institute for Immunology Postfach Basel CH-4005 Switzerland

**Keywords:** Lymphoma, *Xenopus*, Tumor transplantation

## Abstract

Three new lymphoid tumors offering an assortment of variants in terms of MHC class I expressions, MHC class II expression, and Ig gene transcription have been discovered in the amphibian *Xenopus*. One was developed in an individual of the isogenic LG15 clone (LG15/0), one in a frog of the LG15/40 clone (derived from a small egg recombinant of LG15), and one (*ff*-2) in a male *ff* sib of the individual in which MAR1, the first lymphoid tumor in Xenopus was found 2 years ago. These tumors developed primarily as thymus outgrowths and were transplantable in histocompatible tadpoles but not in nonhistocompatible hosts. Whereas LG15/0 and LG15/40 tumor cells also grow in adult LG15 frogs, the *ff*-2 tumor, like the MAR1 cell line, is rejected by adult *ff* animals. Using flow cytometry with fluorescence-labeled antibodies and immunoprecipitation analysis, we could demonstrate that, like MAR1, these three new tumors express on their cell surface lymphopoietic markers recognized by mAbs FIF6 and RC47, as well as T-cell lineage markers recognized by mAbs AM22 (CD8-1ike) and X21.2, but not by immunologobulin (Ig) nor MHC class II molecules. Another lymphocyte-specific marker AM15 is expressed by 15/0 and 15/40 but not *ff*-2 tumor cells. The *ff*-2 tumor cell expresses MHC class molecule in association with *β*2-microglobulin on the surface, 15/40 cells contain cytoplasmic I *α* chain that is barely detected at the cell surface by fluocytometry, and 15/0 cells do not synthesize class I *α* chain at all. The three new tumors all produce large amounts of IgM mRNA of two different sizes but no Ig protein on the membrane nor in the cytoplasm. All tumor cell types synthesize large amount of Myc mRNA and MHC class I-like transcripts considered to be non classical.


*Developmental Immunology*, 1994, Vol 3, pp. 297–307
Reprints available directly from the publisher
Photocopying permitted by license only

© 1994 Harwood Academic Publishers GmbH
Printed in Singapore

### Lymphoid Tumors of *Xenopus laevis* with Different Capacities for Growth in Larvae and Adults

JACQUES ROBERT,*† CHANTAL GUIET,† and LOUIS DU PASQUIER†

†*Basel Institute for Immunology, Postfach, CH-4005 Basel, Switzerland*

Three new lymphoid tumors offering an assortment of variants in terms of MHC class I expressions, MHC class II expression, and Ig gene transcription have been discovered in the amphibian *Xenopus*. One was developed in an individual of the isogenic LG15 clone (LG15/0), one in a frog of the LG15/40 clone (derived from a small egg recombinant of LG15), and one (*ff*-2) in a male *ff* sib of the individual in which MAR1, the first lymphoid tumor in *Xenopus* was found 2 years ago. These tumors developed primarily as thymus outgrowths and were transplantable in histocompatible tadpoles but not in nonhistocompatible hosts. Whereas LG15/0 and LG15/40 tumor cells also grow in adult LG15 frogs, the *ff*-2 tumor, like the MAR1 cell line, is rejected by adult *ff* animals. Using flow cytometry with fluorescence-labeled antibodies and immunoprecipitation analysis, we could demonstrate that, like MAR1, these three new tumors express on their cell surface lymphopoietic markers recognized by mAbs F1F6 and RC47, as well as T-cell lineage markers recognized by mAbs AM22 (CD8-like) and X21.2, but not by immunologobulin (Ig) nor MHC class II molecules. Another lymphocyte-specific marker AM15 is expressed by 15/0 and 15/40 but not *ff*-2 tumor cells. The *ff*-2 tumor cell expresses MHC class I molecule in association with $\beta$2-microglobulin on the surface, 15/40 cells contain cytoplasmic class I $\alpha$ chain that is barely detected at the cell surface by fluocytometry, and 15/0 cells do not synthesize class I $\alpha$ chain at all. The three new tumors all produce large amounts of IgM mRNA of two different sizes but no Ig protein on the membrane nor in the cytoplasm. All tumor cell types synthesize large amount of Myc mRNA and MHC class I-like transcripts considered to be non classical.

KEYWORDS: Lymphoma, *Xenopus*, Tumor transplantation.

### INTRODUCTION

The embryonic and larval development of the anuran amphibian *Xenopus* is a good system for studying ontogenic aspects of the immune system (review in Flajnik et al., 1987; Du Pasquier et al., 1989). Moreover, the unique developmental transition occurring during metamorphosis allows one to study the regulation of the immune fuction in a way that is not possible in mammals. During this crisis period, the immune system undergoes a major remodeling: MHC class I antigens appear on cell membranes (Du Pasquier et al., 1979; Flajnik et al., 1986; Flajnik and Du Pasquier, 1988); the tissue distribution of MHC class II changes (Du Pasquier and Flajnik, 1990); the adult antibody repertoire replaces the larval one (review in Du Pasquier et al., 1989); and an active tolerance to newly arising, adult-specific antigens is acquired (Du Pasquier and Bernard, 1980). However, the advantages of this model have been counterbalanced by a limited armamentarium of methods for studying the phenomena associated with the metamorphosis of the *Xenopus* immune system. This area of research would greatly benefit from the development of new models such as cell lines and clones with larval or adult phenotypes and exhibiting stage-specific responses *in vitro*. Lymphoid cell lines are exceedingly rare in amphibians. In fact only one such line has been described so far; B3B7 is derived from a thymic lymphoid tumor MAR1 and grows in histocompatible *ff* tadpoles but not in histocompatible *ff* adults (Du Pasquier and Robert, 1992). Recently, three new, apparently similar tumors developed in our colony, each in a different strain. We report here our characterization of these tumors by *in vivo*

*Corresponding author.

transplantation experiments, flow cytometry, immunoprecipitation, and Western and Northern blot analysis. In future experiments on the metamorphosis of the immune system, we hope to be able to exploit the differences between adult and larval behavior that we found.

### RESULTS

### Origin of Lymphoid Tumors

The three new lymphoid tumors were found in our frog colony during August and September 1992. They developed primarily in the thymus, and the frogs bearing a large outgrowth on one side of the head were easily detected. In each case, samples of the tumors' tissue and cell suspension were frozen, transplanted in other animals, and put into culture medium in an attempt to grow the cell *in vitro*.

### Transplantation Studies

The *ff*-2 tumor appeared in a male *ff*, sib of the frog in which the first lymphoid tumor, MAR1, was detected in December 1991 (Du Pasquier and Robert, 1992). The animal carrying this tumor was kept alive as long as possible, and at the time of sacrifice, two months later, there were multiple metastases in liver, kidney, spleen, and blood. The contralateral thymic lobe was not affected by the tumor. Cells of this tumor are transplantable in partially inbred MHC-homozygous tadpoles but not in unrelated larval hosts (Table 1). Ascites containing about $10^7$ tumor cells are usually obtained 2–3 weeks after intraperitoneal injection of a $5 \times 10^4$–$1 \times 10^5$ single tumor cell suspension. Like MAR1 cells, *ff*-2 cells are rejected by postmetamorphic young or fully grown adults of the same family. Dorsal subcutaneous injections of $1 \times 10^6$ cells, which in our experience is the most sensitive route of injection in adults, failed to produce any tumor. Rejection occurs as soon as 1 month after the completion of metamorphosis. Both in normal and sodium perchlorate blocked tadpoles (that do not metamorphose), metastases developed secondarily in spleen, liver, and at the site of injection. In one case, metastases were also observed in kidney.

The other two tumors appeared in two LG15 lines—one in the original LG15/40, a clone derived from a LG15 small egg recombined clone. Both 15/0 and 15/40 tumor cells are transplantable in isogenic

TABLE 1
Results of Transplantation Experiments with the Different Tumor Cell Types

| Tumor | Cell no. ($\times 10^6$) | Injection route | Recipient MHC | Stage | No. of animals | Result |
|---|---|---|---|---|---|---|
| *ff*-2 | 0.5 | i.p. | *f/f* | Tadpole (st. 56–58) | 100 | Ascites, metastasis |
| | 0.5 | i.p. | *f/f* | Blocked tadpole | > 100 | Ascites, metastasis |
| | 1.0 | s.c. | *f/f* | 4-year-old adult | 4 | No growth |
| | 0.5 | i.p. | *a/c* (*LG15*) | Tadpole (st. 56–58) | 5 | No growth |
| 15/40 | 0.5 | i.p. | *a/c* (LG15) | Tadpole (st. 56–58) | 50 | Ascites, metastasis |
| | 0.5 | i.p. | *a/c* (LG15) | Blocked tadpole | 50 | Ascites, metastasis |
| | 1.0 | s.c. | *a/c* (LG15) | 4-year-old adult | 10 | Subcutaneous tumor at site of injection, metastasis |
| | 1.0 | i.p. | *a/c* (LG15) | 4-year-old adult LG15 | 5 | Ascites, metastasis |
| | 0.5 | i.p. | *f/f* | Blocked | 10 | No growth |
| 15/0 | 0.5 | i.p. | *a/c* (LG15) | Tadpole (st. 56–58) | 20 | Ascites, metastasis |
| | 0.5 | i.p. | *a/c* (LG15) | Blocked tadpole | 20 | Ascites, metastasis |
| | 2.0 | s.c. | *a/c* (LG15) | 4-year-old adult | 7 | Subcutaneous tumor at site of injection, metastasis |
| | 0.5 | i.p. | *f/f* | Blocked | 10 | No growth |

LG15 tadpoles but not in allogenic hosts (Table 1). Three weeks after intraperitoneal injection, tumor cells were detected as ascites and in metastases. However, the 15/0 and 15/40 tumor cells differ strikingly from MAR1 and *ff*-2 in that they also grow well in 4-year-old LG15 adults. Dorsal subcutaneous injections of $2 \times 10^6$ cells produce a large solid tumor containing more than $10^9$ cells after one month, usually in the space beneath the muscles and skin at the site of injection. Metastases developed secondarily in spleen, liver, and kidney. The 15/40 tumor cells transplanted intraperitoneally also grow albeit at a somewhat slower rate, and after 2 months, ascites and metastases in the spleen were found. Allogenic adults rejected cells from both tumors.

In order to check for the presence of infectious tumor viruses, sonicated supernatants of 15/40 and MAR1 tumor cells were injected after filtration through 22-$\mu$m membranes into histocompatible larval and adult hosts. Injected tadpoles metamorphosed normally, and even after 1 year, no detectable tumors developed. The 15/40 tumor has been adapted *in vitro* with essentially the same protocol as the one described for MAR1 (Du Pasquier and Robert, 1992).

### Characterization of the Cell Surface by Flow Cytometry

The expression of cell-surface antigens was investigated by flow cytometry after staining with fluorescence-labeled monoclonal antibodies. Table 2 summarizes the results. Some common features are shared by all tumor cell types; all of them, like the previously described MAR1 cell line, are strongly stained for T-cell markers recognized by mAb AM22 (which is specific for a putative CD8 equivalent of *Xenopus*) and X21.2, as well as for the markers of the lymphopoietic lineage recognized by F1F6 and RC47. None of the MHC class II-specific mAbs, nor Ig-specific mAbs, including anti-$\mu$, anti-$\upsilon$, anti-$\chi$, anti-Ig light chain, and anti-$V_H$, stain the cell surface of 15/0 and *ff*-2 tumors. However, MHC class II-specific mAbs stained 15/40 very weakly; it is not yet clear whether this represents nonspecific staining or a very weak expression of MHC class II molecules. All the Ig-specific mAbs are negative with the 15/40 tumor cells.

Other features discriminate among the tumors. The class I-specific TB17 mAb positively stains *ff*-2 cells (Fig. 1); because all live cells are stained, this is not the result of contamination from host cells. In

TABLE 2
Flow Cytometry Analysis of the Different Tumor Cell Types

| Monoclonal antibodies | | Specificity | References[a] | Adult lymphocytes: Spleen | Thymus | Tumor cells: B3B7 | *ff*-2 | 15/0 | 15/40 |
|---|---|---|---|---|---|---|---|---|---|
| Anti-MHC | TB17 | Class I (44–45 kd) | 4 | + +[b] (> 90%) | + + (> 95%) | − | + | − | − |
| | AM20 | Class II (30 kd) | 5 | + + (> 90%) | + + (> 90%) | − | − | − | ± |
| | 14A2 | Class II (30 kd) | 5 | + + (> 90%) | + + (> 90%) | − | − | − | ± |
| Anti-T cell | AM22 | CD8-like (35 kd) | 6 | + + (30–50%) | + + (> 50–70%) | + + | + + | + + | + + |
| | X21.2 | T cell (110 kd) | 7 | + (10–30%) | + (90–95%) | + | + | + | + |
| | AM15 | T cell popul. (18 kd) | 5 | + (75–80%) | + (> 90%) | − | − | + + | + |
| Anti-leucocytes | F1F6 | leucocyte, erythrocyte | 8 | + (80–90%) | + (> 90%) | + + | + + | + + | + |
| | RC47 | leucocyte | 6 | ND | + (> 90%) | + | + ND | + | + |
| Anti-Ig | 10A9 | IgM (60–62 kd) | 1 | + + (15–30%) | + (5–10%) | − | − | − | − |
| | 11D5 | IgY (64 kd) | 1 | + (0.5–1%) | + | − | − | − | − |
| | 410D9 | IgX (66 kd) | 2 | + (0.5–2%) | + | − | − | − | − |
| | 13B2 | IgL1 (25 kd) | 3 | + + (2–8%) | ND[c] | − | − | − | − |
| | 1E9 | IgL2 (29 kd) | 3 | + (5–10%) | ND | − | − | − | − |
| | 409B8 | IgL3 (27 kd) | 2 | + + (5–20%) | ND | − | − | − | − |
| | 10C10 | IgVHa | 1 | + + (7–30%) | ND | − | − | − | − |
| | 14B4 | IgVHb | 1 | + + (5–10%) | ND | − | − | − | − |
| | 14D5 | IgVHc | 1 | + + (2–10%) | ND | − | − | − | − |

[a]References:
1. Hsu and Du Pasquier, 1984
2. Hsu and Du Pasquier, unpublished
3. Hsu et al., 1991
4. Flajnik et al., 1991
5. Flajnik et al., 1990
6. Du Plasquier and Flajnik, 1991
7. Nagata, 1985
8. Flajnik et al., 1988

[b]+ + and + relate to the relative brightness of the whole tumor cell population.
[c] ND = not done.

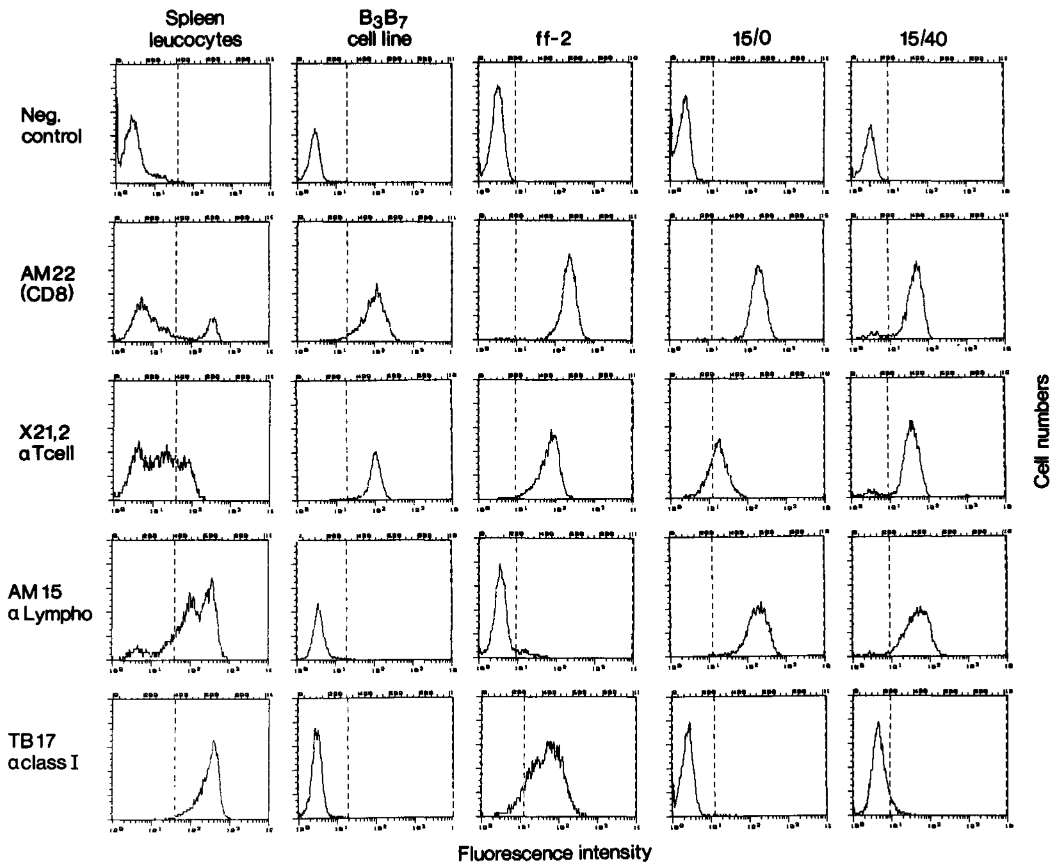

FIGURE 1. Flow cytometry analysis of normal spleen lymphocytes and the various tumors. The specificity of the monoclonal antobodies is given in Table 2.

the case of 15/40 tumor cells, there is a slight shift of the peak with the TB17 mAb that could indicate a low expression of class I molecules at the cell surface (see Western Blot Analysis). The cells from the other tumors are not stained by TB17. Another antibody (AM 15), which stains a lymphocyte subset (Flajnik et al., 1990) more intensively, stains only 15/40 and 15/0 tumor cells, the latter more intensely. The flow cytometric profile obtained with normal spleen cells is usually bimodal with this antibody. The peak stained most intensely is thought to correspond to T cells, because it disappears after thymectomy (J. D. Horton, personal communication). The relative intensity of staining with the general leukocyte marker F1F6 is significantly less with 15/40 cells than with normal lymphocytes or B3B7 cells (data not shown). Comparable profiles of each tumor type were obtained several times, both with samples of the original tumor and with transplanted tumor cells. The surface expression profile of 15/40 tumor cells was conserved after culture *in vitro*.

### Characterization of the Cell Surface by Immunoprecipitation

In order to confirm the analysis by flow cytometry, we used immunoprecipitation after cell-surface iodination (Fig. 2). From lysates of all tumor cell types, including the B3B7 cell line, a unique band, which corresponds to a band of 36 kd in normal splenocyte lysates, was detected with mAb AM22 on SDS-PAGE gels under reducing conditions (Fig. 2B). A second band of 38 kd, usually present in normal spleen lysates (Fig. 2A), could not be found even when the cells were preincubated with the mAb before lysis (data not shown); this result suggests that these tumor cells express only one chain of the putative. CD8 molecule.

With the other T-cell-specific marker recognized by mAb X21.2, the situation is more complex (Fig. 2B). Whereas three bands are immunoprecipitated from the B3B7 cell line, only two bands are present in lysates of *ff*-2, and no signal at all could be

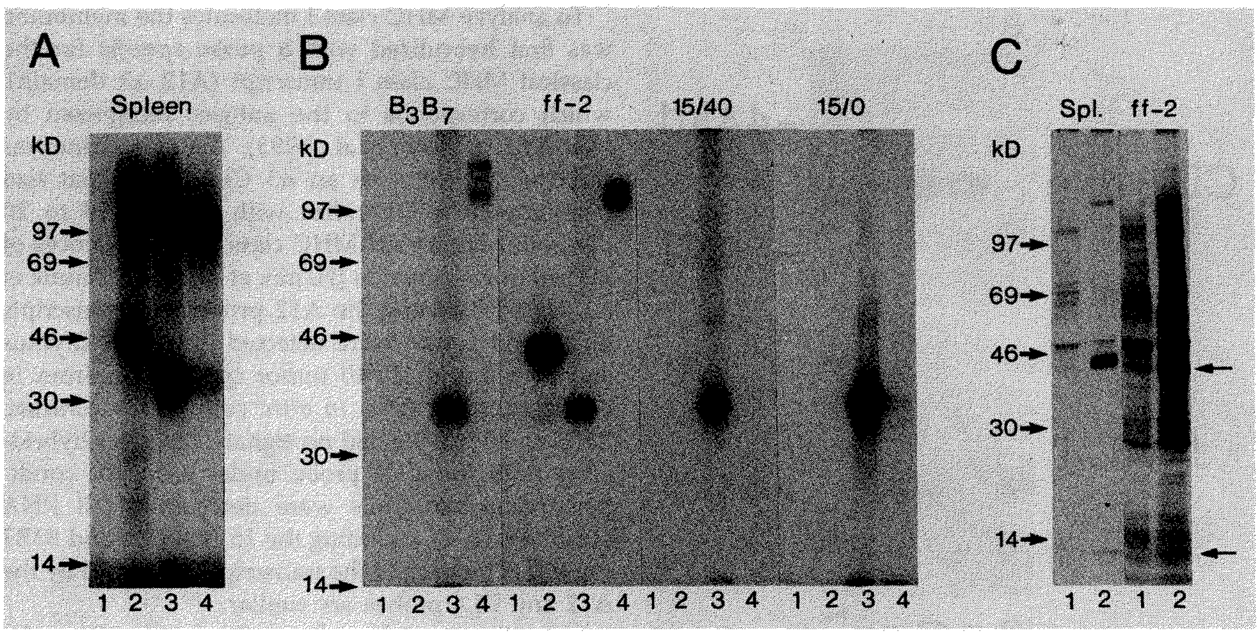

FIGURE 2. Comparative immunoprecipitation analysis under reducing conditions of cell-surface markers expressed by normal lymphocytes and the various tumors. (A and B) Surface iodinated lysate from normal outbred lymphocytes and each tumor cell type were immunoprecipitated by the SPIT technique and separated on 10% SDS-PAGE gels. Lane 1: Negative control with nonspecific mAb supernatants, Lane 2: TB17 anti-MHC class I, Lane 3: AM22 anti-CD8-like, Lane 4: X21.1 anti-T cell. Only one band is present in 15/0 lysate. (C) Normal lymphocytes and *ff*-2 tumor cells biosynthetically labeled with $^{35}S$-methionine were preincubated with (1) normal nonimmune *Xenopus* serum or (2) specific anti-*ff* alloserum, lysed, immunoprecipitated by protein A, and loaded on a 7.5–15% SDS-PAGE gel gradient.

detected with lysates of 15/40 and 15/0 tumor cells, even when more gentle washing conditions are used, and despite the fact that a signal was recorded in immunofluorescence. Normal spleen lymphocytes express one major band, but two faint bands were also detected after a long exposure (Fig. 2A).

Using the class-I-specific mAb TB17, we precipitated a strong band of 45 kd corresponding to a class I $\alpha$ chain from cell-surface iodinated *ff*-2 but not the other tumors. As previously reported, $\beta$2-microglobulin (the MHC class I light chain) could not be detected using this mAb (Flajnik et al., 1991). When we used a specific anti-*ff* alloserum preadsorbed before lysis and immunoprecipitation on metabolically labeled *ff*-2 tumor cells or normal splenocytes, a band of 13 kd corresponding to $\beta$2m was clearly seen; this band was not present in controls with nonimmune *Xenopus* serum (Fig. 2C). A higher background is allways obtained after immunoprecipitation of tumor cell lysates with protein A, even after preincubation with normal *Xenopus* serum and protein A.

No Ig molecules were detected from iodinated cell-surface protein of any tumor cell (data not shown).

### Western Blotting

When cell lysates were analyzed by Western blotting, we could identify the MHC class I $\alpha$ chain protein in lysates of the 15/40 tumor (Fig. 3). To avoid contamination with host class I, lysates were prepared from tumor cells grown *in vitro*. A band with a slightly slower mobility than that of *ff*-2 tumor cells was clearly present, and no signal was detected in lysates of 15/0 tumor cells or B3B7. This difference in gel mobility is probably due to allelic differences between the LG15 and *ff* strains of *Xenopus* (Flajnik et al., 1991). The integrity of cell lysates was controlled with mAb AM22, which stained a band of the expected apparent molecular weight in all tumor samples, but not in spleen. We interpret these findings to mean that MHC class I $\alpha$ chain is present at least in the cytoplasm of 15/40 tumor cells.

No Ig could be detected by this Western blotting. Additional experiments to check for the presence of IgM in the cytoplasm by metabolic labeling and by immunofluorescence microscopy on fixed tumor cells were all negative (data not shown).

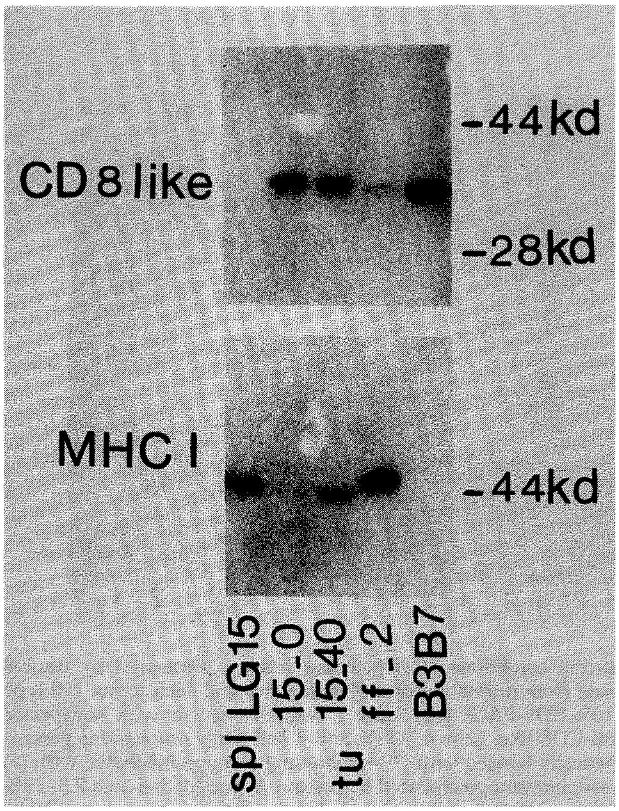

FIGURE 3. Western blot analysis by chemoluminescence assays of CD8-like and MHC class I molecules under reducing conditions from normal spleen lymphocytes and each tumor cell type.

### Northern Blotting

Although cells of the three new tumors do not express Ig molecules on the cell surface or in the cytoplasm, they all produce large amounts of $\mu$ heavy-chain mRNA detectable on Northern blots; no signal at all could be detected in the B3B7 cell line (Fig. 4). In all three new tumors, the C$\mu$ probe detects, in addition to the usual 1.8-kb $\mu$ mRNA previously described (Schwager et al., 1988), another transcript of 1.35 kb that is barely detectable in normal lymphocytes. The shorter $\mu$ band was observed in several experiments, and it does not seem to result from a general RNA degradation, as judged by hybridization of other probes on the same membrane. The large amount of specific $\mu$ messenger cannot be attributed to contamination from host RNA alone. Moreover, RNA from 15/40 cells grown *in vitro* contains comparably large amount of $\mu$ transcript.

To analyze MHC class I molecules the membrane was first hybridized with a probe specific for the classical MHC class I transcript (A12 $\alpha$3 domain), which corresponds to the antigen recognized by mAb TB17 (Schum et al., 1993). The membrane was then hybridized with an $\alpha$3 CL4 probe that had been shown to hybridize with at least 10 to 20 different nonclassical MHC class I-like sequences of yet unknown function (Flajnik et al., 1991; Flajnik et al., 1993). By using the A12 probe, two transcripts of 2.4 and 1.4 kd were detected in normal thymus and in *ff*-2 and 15/40 tumor cells (either from *in vivo* transfer or from *in vitro* culture). In contrast, B3B7 and 15/0 yielded no signals. But after hybridizing with the CL4 probe under stringent conditions, two transcripts were detected in all RNA samples (Fig. 4), including the 15/0 tumor and B3B7 cell line. The sizes of the transcripts identified by the A12 and CL4 probes are similar.

Hybridization with a *Xenopus myc*-specific probe detected a large amount of Myc mRNA, especially in the B3B7 cell line and the 15/0 tumor, which we estimate to contain more Myc RNA than do normal thymocytes (Fig. 4) or the A6 kidney cell line (data not shown). Two distinct copies of *Xenopus* c-*myc* gene have been isolated, but their discrimination is only possible by using oligonucleotide specific of the 5′ untranslated region (Principaud and Spohr, 1991; Vriz et al., 1989). The elevated or deregulated expression of c-*myc* oncogene has been reported for a large number of tumor cells in higher vertebrates and its involvement in neoplastic transformation has been postulated (for a review, see DePinho et al., 1991). A similar implication of c-*myc* is likely in these *Xenopus* lymphoid tumors.

### Discussion

We regularly examine our frogs for evidence of tumors and found no lymphoid tumors in the first 20 years or so of its existence. Then, at the beginning of 1992, the first *Xenopus* lymphoid tumor MAR1 was discovered in our colony (Du Pasquier and Robert, 1992), and 8 months later, three more appeared. We are not yet sure of the factors responsible for these neoplasms. It would not seem to be genetics, for three different clonal lines of *Xenopus* are involved (*ff*, LG15, and LG15/40). Moreover, infectious fungi or bacteria are definitively not involved. The new tumors, like MAR1, can only be propagated in isogenic or highly homozygous hosts;

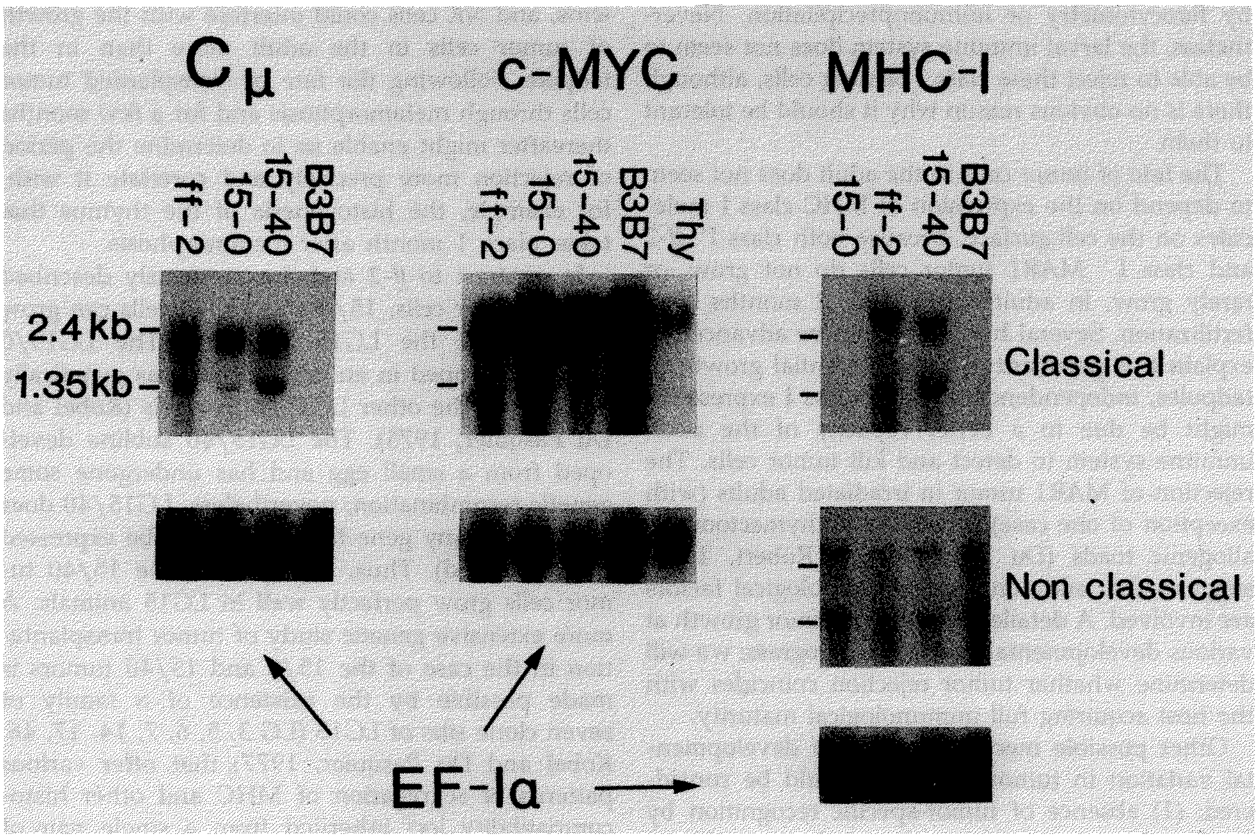

FIGURE 4. Northern blot analysis of the various tumors and normal thymocytes (Thy) with *Xenopus* Cμ, *c-myc*, and classical and nonclassical MHC class I probes. Cytoplasmic RNA (10 μg) was electrophoresed in a 1% formaldehyde-agarose gel, transfered to a nylon membrane and hybridized with $^{32}$P-labeled probes. The sizes of mRNAs were calibrated using RNA markers from BRL. The integrity of each RNA sample was controlled by reprobing the membrane with a *X. laevis* Ef-1α probe.

15/40 tumor cells can also grow *in vitro*. Such criteria strongly support the interpretation that we are dealing with real lymphoid tumors and not with granulomas (Asfari and Thiébaud, 1988). Each tumor seems to be the result of an independent oncogenic event. That is, they appeared in animals of different genetic background, they behave differently after transplantation, and they display distinct phenotypes, as shown by flow cytometry and Northern blotting. All attempts to demonstrate the presence of a virus have been unsuccessful. Like the B3B7 cell line, which was derived from the MAR1 tumor, all of the new tumors have mixed T-B phenotypes (several surface markers of T cells, but Ig RNA is transcribed). Aside from the possibility of enabling us to learn more about neoplasm biology in amphibians, these tumors provide a panel of new experimental models to study lymphocyte differentiation and the ontogeny of the immune system in amphibians.

### Growth Capacities of the Transplanted Tumor Cells in Larval and Adult Histocompatible Hosts

MAR1 and *ff*-2 tumor cells grow only in histocompatible larval hosts, whereas 15/0 or 15/40 tumor cells grow both in larvae and adults. The *ff*-2 tumor cells express clasical MHC class I α chain in association with β2-microglobulin on the cell surface, whereas MAR1-derived B3B7 cells of the same strain (*ff*) do not. In contrast to mammals, which express class I in the embryonic stages, the classical MHC class I molecules of *Xenopus* (present on the membrance of almost all cells in the adult) are not detected in young tadpoles and appear only during metamorphosis (Flajnik et al., 1986; Flajnik and Du Pasquier, 1988). *In vivo* transplantation into young tadpoles (stages 55–56), or into tadpoles that have been inhibited from metamorphosing, does not modify class I expressing *ff*-2 tumor cells as judged by fluocytometry or immunoprecipitation. Nevertheless, the larval immune system does not seem to be able to reject these class I bearing cells, although there is no obvious reason why it should be tolerant to them.

The fate of tumor cells in the adult does not seem to depend on the expression of MHC class I molecules on the cell surface, because both class $I^+$ *ff*-2 and class $I^-$ MAR1 tumor cells do not grow, or rarely grow, in adults older than 2 months after fertilization. Several hypothesis can be advanced to explain this peculiarity. The preferential growth in tadpoles, independence of MHC class I expression, might be due to a better capacity of the adult immune system to detect and kill tumor cells. The rejection of MAR1 tumor in irradiated adults (with exception of one case) as well as in thymectomized allogenic toads (Du Pasquier and Robert, 1992) suggests that additional, nonimmunological factors are involved. A detailed analysis of tumor growth at various developmental stages is in progress; we will determine whether tumor rejection coincides with the host acquiring full immunological maturity.

Other possible mechanisms of the developmental variation in tumor rejection should be considered: (1) absence of tumor-specific recognition by the larval immune system, (2) developmental activation in the host of a resistance gene during metamorphosis, and (3) upregulation of expression by the tumor of a new polymorphic determinant acting as a strong antigen recognizable only by the adult TCR or Ig. The presence of other related MHC class I transcripts found in the cytoplasm of 15/0 and B3B7 cell line should also be considered. There exist at least 20 different genes encoding class I-like sequences, most of which are not MHC-linked (Flajnik et al., 1993). Their function and their specific expression are as yet unknown, but it is possible that at least one of these MHC I-like proteins is involved in the developmentally regulated tumor rejection. Within the *ff* family, skin grafts are often accepted for more than 200 days, but these grafts are eventually rejected after 300 days; thus, this family is not isogenic at all histocompatibility loci (Du Pasquier and Chardonnens, 1975). New family studies are necessary to determine whether or not minor histocompatibility antigens still segregating in our *ff* pool are responsible for the rejection of tumor transplants by adults. In any case, we do not understand why the tumor cells should not be accepted for at least some months. In addition to classical immunological parameters, endocrine factors, homing problems, and NK cells could interfere with the growth of tumor cells in the adult more than in the tadpole. Following the fate of transplanted tumor cells through metamorphosis and for a few months thereafter might enable us to determine the period of rejection more precisely and correlate it with, for example, the histogenesis of the thymus that takes place 1 month after metamorphosis.

In contrast to *ff*-2 and the previously described MAR1 tumor cells, 15/0 and 15/40 cells can grow in adults of the LG15 genotype. The LG15/0 tumor developed in an animal that was genetically identical to the other LG15 individuals (Kobel and Du Pasquier, 1975). The LG15/40 subline developed from a small egg and has undergone some genetic recombination; nevertheless; LG15/40 does not express any gene that would not be expressed in LG15 (Ibid). Thus, as expected, the 15/40 tumor cells grow perfectly well in LG15 animals. A more extensive genetic study of tumor transplantation in the case of the 15/0 and 15/40 tumors is made possible by the existence of a family of seven clone sibs of LG15 (LG 3, 5, 6, 7, 14, 17, 46; Kobel and Du Pasquier, 1977) that offer various patterns of segregation of MHC and other histocompatibility loci inherited from a single pair of parents.

### Expression of the Immunoglobulin $\mu$ Messenger

Although the strong expression of $\mu$ Ig heavy-chain messenger by the three new tumors, 15/0, 15/40, and *ff*-2, there is not trace of Ig protein chains. Because specific transcripts of the expected size (Schwager et al., 1988) are present, at least one Ig havey-chain locus must have undergone somatic rearrangement. Experiments are in progress to substantiate and extend this point. In the MAR1 cell line, both Ig heavy-chain and two light-chain loci have been rearranged (Du Pasquier and Robert, 1992; Du Pasquier et al. in preparation). Preliminary investigation with 15/40 tumors cells using specific $V_H$ probes indicates the presence of complete $\mu$ mRNA (data not shown) but does not exclude the synthesis of an additional, truncated messenger as described earlier. Because the translated product might be rather unstable, it would be interesting to submit 15/40 cells to short pulses of metabolic labeling before immunoprecipitation. Anchored or single-sided PCR with a $C\mu$ primer may also be considered in order to isolate and sequence mRNAs of different length (Troutt et al. 1992).

Despite the rearrangement of Ig genes in all tumors, and the expression of Ig messengers in three of them, none of the tumor seems to synthesize Ig protein chains. Perhaps all these messengers are simply out of frame. But another possibility would be that Ig production is selected against, and Ig-negative cells outgrow, the Ig-positive cells in incipient tumors. There might also be some incompatibilities between the expression of the various T-cell markers and the synthesis of Ig. In any case, sequence analysis of corresponding Ig gene rearrangements that are in progress would clarify these point.

### Use of Tumor Cells as a Source of Proteins of Immunological Interest

Large amounts of proteins of immunological interest can be purified from tumor tissue and characterized without contamination by other cell types. One such protein is $\beta2$ microglobulin, for which no specific antibodies are available and for which the gene has not been cloned and sequenced in *Xenopus*. Its association with the class I $\alpha$ chain at the surface of *ff*-2 cells means that anti-MHC I specific allosera can be used to immunoprecipitate and isolate the molecule for peptide sequencing. The tumor offers a better starting material than red cells, the lysate of which are always rich in the globin 13 kd monomer that interferes with $\beta2$-m isolation.

Another protein of interest is the putative CD8 molecule recognized by mAb AM22. The presumption of homology of this molecule to mammalian CD8 is based on the similarity of apparent molecular weight and tissue distribution; there are no protein or DNA sequences. The high level of expression by the various tumors of at least one CD8-like subunit should enable us to immunoprecipitate enough material to sequence.

The 15/40 tumor cells are now proliferating *in vitro*, and new, cloned cell lines will soon be established. The same strategy of successive passages from *in vivo* transplantation to *in vitro* culture is being followed with the two other tumor types.

### MATERIAL AND METHODS

### Toads

The *Xenopus ff* inbred family (Du Pasquier and Chardonnens, 1975) is highly homozygous. As these animals retain skin grafts for more than 200 days, they are considered to be histocompatible. LG15 isogenic clones have been described in detail elsewhere (Kobel and Du Pasquier, 1975). The LG15/40 line arose from the development of a small egg that did not undergo genomic duplication and was therefore recombinant (Kobel and Du Pasquier, 1977; Wilson et al., 1992). Development was blocked before metamorphosis at stage 48 (Nieuwkoop and Faber, 1967) by adding 0.1% potassium perchlorate ($KClO_4$) to the water (Buscaglia, 1977).

### Cell Culture

A cell line was derived from the LG15/40 tumor in the same way as line B3B7 was derived from MAR1 (Du Pasquier and Robert, 1992) by 6 months of repeated *in vivo* and *in vitro* passage. Culture media and culture conditions have been described (Du Pasquier and Robert, 1992).

### Flow Cytometry

Samples of $10^5$ cells stained with fluorescence-labeled hybridoma supernatants were analyzed by flow cytometry on a FACScan apparatus, as described by Du Pasquier and Flajnik (1990).

### Immunoprecipitation

Cell-surface iodination and lysis in NP-40 were done as described (Kaufman et al., 1985; Flajnik and Du Pasquier, 1988). A solid-phase immunoisolation technique (SPIT) was used (Tamura et al., 1984; Flajnik et al., 1988). A 96-well plate was coated overnight at 4°C with 100 $\mu$l of goat anti-mouse Ig at 100 $\mu$g/ml (Kierkegaard), washed and blocked with 1% BSA in PBS, and incubated at room temperature 2 × 2 hr with 100 $\mu$l of specific hybridoma supernatants. After washing several times, radiolabeled cell-surface lysates ($10^8$ cpm/ml corresponding to ca. $10^6$ cells ) were incubated overnight at 4°C. After washing twice with cold 50 mM Tris (pH 8), 500 mM NaCl, 1 mg/ml ovalbumin, and twice with 50 mM Tris, 100 mM NaCl, the samples were resuspended in Laemlli sample buffer with 5% 2-mercaptoethanol and separated on 10% SDS-polyacrylamide gels. Cells labelled *in vitro* with $^{35}$S-methionine were immunoprecipitated with specific alloantisera, as described (Kaufman et al., 1985; Flajnik and Du Pasquier, 1988).

### Western Blot Analysis

Cell lysates were prepared with NP-40, as described (Flajnik et al., 1991), separated on 10% SDS-polyacrylamide gels, and electroblotted on immunlite membrane (Bio Rad). After incubation with hybridoma supernatants, the membranes were assayed by chemoluminescence (Bio Rad).

### Northern Blot Analysis

Cytoplasmic RNA from a cell suspension of spleen lymphocytes and cell lines was isolated by the vanadyl-ribonuclease complex method (Berger, 1987), and liver total RNA was isolated by the guanidinium isothiocyanate procedure (Chomcyznski and Sacchi, 1987). RNA (10 $\mu$g/slot) was separated on 1% agarose–2.2 M formaldehyde gel according to a standard protocol (Davis et al., 1986), transferred to Hybond-N+ membranes (Amersham), and fixed with 50 mM NaOH. Prehybridization (6–16 hr) and hybridization (24–48 hr) were performed in 50% formamide at 45°C with $^{32}$P-labeled *Xenopus* C$\mu$ or $V_H$ probes (Schwager et al., 1988). MHC class I classical and nonclassical $\alpha$3 probes were a kind gift of M. Flajnik (Shum et al., submitted for publication; Flajnik et al., 1991). The *myc* probe was kindly provided by E. Principaud (Principaud and Spohr, 1991). Filters were washed 2× 30 min at 65°C in 0.2× SSC. 0.2% SDS. The sizes of mRNAs were calibrated using RNA markers (BRL). The integrity of each RNA sample was controlled by reprobing the membrane with a *X. laevis* elongation factor 1$\alpha$(Ef-1$\alpha$) probe (Krieg et al., 1989)

### ACKNOWLEDGMENTS

We wish to thank Drs. Isabelle Chrétien, Klaus Karjalainen, and Charley Steinberg for helpful suggestions and critical reading of the manuscript. The Basel Institute for Immunology was founded and is supported by F. Hoffman La Roche Ltd., Basel, Switzerland.

*(Received August 25, 1993)*

*(Accepted November 15, 1993)*

### REFERENCES

Asfari M., and Thiébaud G. H. (1988). Transplantation studies of a putative lymphosarcoma of *Xenopus*. Cancer Res. **48:** 954–957.

Berger S. L. (1987). Isolation of cytoplasmic RNA: Ribonucleoside-vanadyl complexes. Method. Enzymol. **152:** 227–234

Buscaglia M. (1977). Dissociation des déterminants des développements gonadiques et somatiques au moment de la métamorphose chez *Xenopus (Xenopus laevis Daud.)*. CR des Séances SPHN Genève **11:** 53–58.

Chomczynski P., and Sacchi N. (1987). Single-step method of RNA isolation by acid guanidinium thiocyanate-phenol-chloroform extraction. Anal. Biochem. **162:** 156–159.

Davis L. G., Dibner M. D., and Battery J. F. (1986). *Basic methods in molecular biology* (New York: Elsevier Science Publishing Co.).

DePinho R. A., Schreiber-Agus N., and Alt F. W. (1991). *myc* family oncogenes in the development of normal and neoplastic cells. Adv. Cancer Res. **57:** 1–46.

Du Pasquier L., and Bernard C. C. A. (1980). Active supression of the allogeneic histocompatibility reactions during the metamorphosis of the clawed toad *Xenopus*. Differentiation **16:** 1–7.

Du Pasquier L., Blomberg B., and Bernard C. C. A. (1979). Ontogeny of immunity in amphibians: Changes in antibody repertoire and appearance of adult MHC antigens in *Xenopus*. Eur. J. Immunol. **9:** 900–906.

Du Pasquier L., and Chardonnens X. (1975). Genetic aspects of the tolerance to allografts induced at metamorphosis in the toad *Xenopus laevis*. Immunogenetics **2:** 431–440.

Du Pasquier L., and Flajnik M. F. (1990). Expression of MHC class II antigens during *Xenopus* development. Dev. Immunol. **1:** 85–95.

Du Pasquier L., and Robert J. (1992). *In vitro* growth of thymic tumor cell lines from *Xenopus*. Dev. Immunol. **2:** 295–307.

Du Pasquier L., Schwager J., and Flajnik M. F. (1989). The immune system of *Xenopus*. Ann. Rev. Immunol. **7:** 251–275.

Flajnik M. F., Canel C., Kramer J., and Kasahara M. (1991). Evolution of the major histocompatibility complex: Molecular cloning of major histocompatibility complex class I from the amphibian Xenopus. Proc. Natl. Acad. Sci. USA **88:** 537–541.

Flajnik M. F., and Du Pasquier L. (1988). MHC class I antigens as surface markers of adult erythrocytes during metamorphosis of *Xenopus*. Dev. Biol. **128:** 198–206.

Flajnik M. F., Ferrone S., Cohen N., and Du Pasquier L. (1990). Evolution of the MHC: Antigenicity and unusual tissue distribution of *Xenopus* (frog) class II molecules. Mol. Immunol. **27:** 451–462.

Flajnik M. F., Hsu E., Kaufman J. F., and Du Pasquier L. (1988). Biochemistry, tissue distribution, and ontogeny of surface molecules detected on *Xenopus* hemopoietic cells. In: Comparative Aspects of Differentiation Antigens in Lymphohematopoeitic Tissues, Miyasaka M., and Trnka Z., Eds. New York: Marcel Dekker, p. 387.

Flajnik M. F., Kasahara M., Shum B. P., Salter-Cid L., Taylor E., and Du Pasquier L. (1993). Identification of a large family of non MHC linked class I genes expressed at the RNA level in the Amphibian *Xenopus*. EMBO J. **12:** 4385–4396.

Flajnik M. F., Kaufman J. F., and Du Pasquier L. (1987). Change in the immune system during metamorphosis of *Xenopus*. Immunol. Today **8:** 58–63.

Flajnik M. F., Kaufman J. F., Hsu E., Manes M., Parisot R., and Du Pasquier L. (1986). Major histocompatibility complex-encoded class I molecules are absent in immunologically competent *Xenopus* before metamorphosis. J. Immunol. **137:** 3891–3899.

Flajnik M. F., Taylor E., and Canel C., Grossberger D., and Du Pasquier L. (1991). Reagent specific for MHC I antigens of *Xenopus*. Amer. Zool. **31:** 580–591.

Hsu E., and Du Pasquier L. (1984). Studies in *Xenopus* immunoglobulins using monoclonal antibodies. Mol. Immunol. **21:** 257–270.

Hsu E., Lefkovits I., Flajnik M., and Du Pasquier L. (1991). Light chain heterogeneity in the Amphibian *Xenopus*. Mol. Immunol. **28:** 985–994.

Kaufman J. F., Flajnik M. F., Du Pasquier., and Riegert P. (1985). *Xenopus* MHC class II molecules: I. Identification and structural characterization. J. Immunol. **134:** 3248–3257.

Kobel H. R., and Du Pasquier L. (1975). Production of large clones of histocompatible, fully identical clawed toads (*Xenopus*). Immunogenetics **2:** 87–91.

Kobel H. R., and Du Pasquier L. (1977). Strains and species of *Xenopus* for immunological research. *In:* Developmental Immunology, Solomon J. B., and Horton J. D., Eds, (Amsterdam, Elsevier North-Holland), pp. 299–306.

Krieg P. A., Varnum S. M., Wormington W. M., and Melton D. A. (1989). The mRNA encoding elongation factor 1-$\alpha$ (Ef-1$\alpha$) is a major transcript at the midblastula transition in *Xenopus*. Dev. Biol. **133:** 93–100.

Nagata S. (1985). A cell surface marker of thymus dependent lymphocytes in *Xenopus laevis* is identified by mouse monoclonal antibody. Eur. J. Immunol. **15:** 837–841.

Nieuwkoop P. D., and Faber J. (1967). Normal table of *Xenopus laevis* (Daudin) (Amsterdam: North Holland).

Principaud E., and Spohr G. (1991). *Xenopus laevis* c-*myc* I amd II genes: molecular structure and development expression. Nucleic Acids Res. **19:** 3081–3088.

Schwager J., Mykoryak C. A., and Steiner L. A. (1988). Amino acid sequence of heavy chains from *Xenopus laevis* IgM deduced from cDNA sequences: Implication for evolution of immunoglobulin domains. Proc. Natl. Acad. Sci. USA **85:** 2245–2249.

Shum B. P., Avila D., Du Pasquier L., Kasahara M., and Flajnik M. F. (1993). Isolation of a classical MHC class I cDNA from an Amphibian. J. Immunol. **151:** 5376–5386.

Tamura G. S., Morris O. D., Gallatin W. M., Mc Grath M. S., Weissman I. L., and Pillemer E. A. (1984). Isolation of molecules recognized by monoclonal antibodies and antisera: the solid phase immunoisolation technique. Anal. Biochem. **136:** 458–464.

Troutt A. B., McHeyzer-Williams M. G., Pulendran B., and Nossal G. J. V. (1992). Ligation-anchored PCR: A simple amplification technique with single-sided specificity. Proc. Natl. Acad. Sci. USA **89:** 9823–9824.

Vriz S., Taylor M., and Mechali M. (1989). Differential expression of two *Xenopus* c-*myc* protooncogenes during development. EMBO J. **8:** 4091–4097.

Wilson M., Marcuz A., Courtet M., and Du Pasquier L. (1992). Sequences of C$\mu$ and the $V_H1$ family in LG7, a clonable strain of *Xenopus*, homozygous for the immunoglobulin loci. Dev. Immunol. **3:** 13–24.

